# Accidental migration of dental implant into the nasal cavity: Spontaneous expulsion through the nose

**DOI:** 10.4317/jced.58427

**Published:** 2021-10-01

**Authors:** José-María Sanchis, José-María Díaz

**Affiliations:** 1 Service of Stomatology and Maxillofacial Surgery. Hospital General Universitario de Valencia. Spain

## Abstract

Implant migration into the nasal fossa is a rare complication and it requires extraction by anterior rhinoscopy. We report a clinical case of placement of short dental implants, fixed or intruded in the nasal fossa floor, which was aspirated by the patient and spontaneously expelled a few days later. To the best of our knowledge, there have been no reports of spontaneously expulsion through the nasal cavity.

** Key words:**Dental implant, nasal cavity, accidental migration.

## Introduction

The possibility of complications due to displacement and migration of dental objects (burs, teeth, surgical instrument breakage, anesthesia needles, prosthetic components, etc.) to adjacent craniofacial structures is not common, although it is well documented in the literature (1). The extensive rise in the use of intraosseous dental implants along with the great popularity of invasive techniques have made these become one of the most frequent causes of migration to unwanted structures (2). One of the most frequent is accidental migration into the maxillary sinus. In 2015, Jeong *et al*. (3) found 49 published cases between 2000 and 2013. The intrusion of dental implants into the maxillary sinus as well as the different techniques for the maxillary sinus floor elevation have caused that the number of cases reported are steadily increasing (4-5). The incidence of dental implant displacement into the maxillary sinus should be much higher than the estimated in the literature.

Although the nasal floor elevation is less frequent, is a technique also used to place dental implants, usually since 2010 (6-9), although not exempt of complications such as alterations of the nasal flow (10), migration into the nasal septum (11) or displacement into the nasal fossa (12).

We report a clinical case of placement of short dental implants, fixed or intruded in the nasal fossa floor, which was aspirated by the patient and spontaneously expelled a few days later.

## Case Report

**Figure 1:**
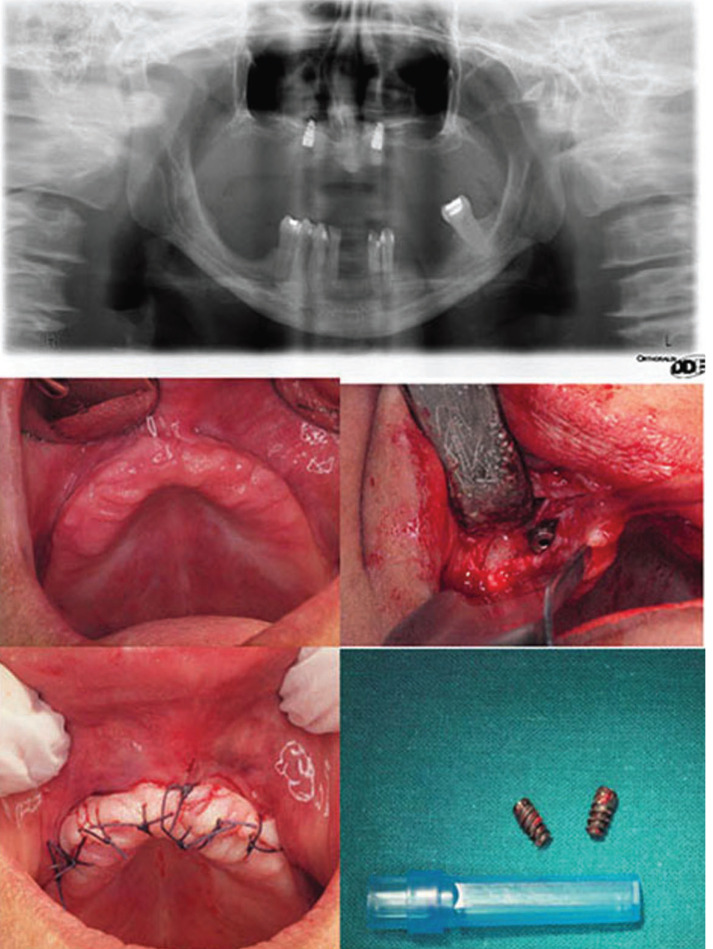
Removal of the implants.

**Figure 2:**
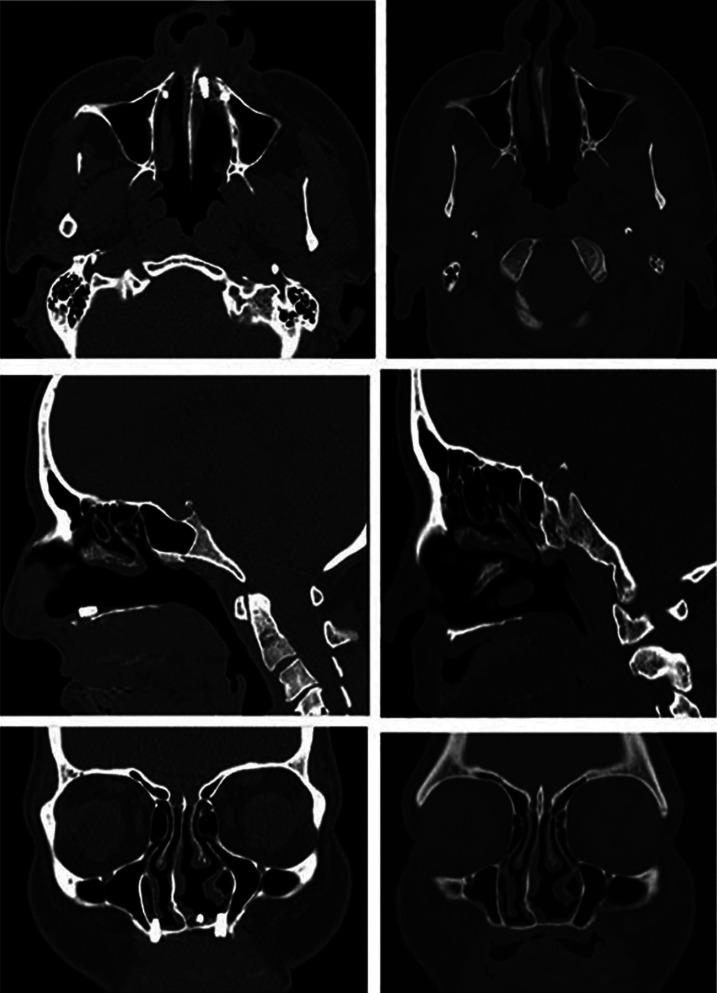
Axial, sagittal and coronal CT scan slices before and after spontaneous expulsion through the nose.

A 41-years-old female with no relevant medical history or allergies known was referred to our service. The patient was treated with 4 short dental implants in the anterior maxilla that were placed in the nasal fossa floor. One year after the surgery, and referring osseointegration problems (one of the implants was lost), the patient visited the otolaryngologist complaining of nasal discomfort. A computed tomography (CT) scan revealed the migration of one of the implants into the nasal fossa. The patient was referred to the Oral and Maxillofacial Surgery Department for the removal of the implants, but during this period, the patient spontaneously expelled the migrated implant through the nose. The two remaining implants were removed to avoid future problems (Figs. 1,2).

## Discussion

The increasing number of implants placed in situations of significant atrophy of the maxilla leads to appearance of different complications, including the migration of the dental implants into adjacent structures as the maxillary sinus or the nasal fossa. Branemark *et al*. (13), in 1984, confirmed in their studies with dogs, the possibility of penetrating with the implants into de maxillary sinus or nasal fossa, without adverse reactions. The perforation or elevation of the maxillary sinus floor and the nasal fossa, with or without bone grafting as well as the direct or indirect elevation has become a common practice in implant dentistry.

The causes of implant migration into the nasal fossa include related to the professional (inexperience or lack of planning), related to the anatomical structures (bone atrophy or bone resorption), related to the prosthesis (early or inadequate prosthetic loading). In our case, the placement of short implants in an atrophic bone, probably associated with changes in the intranasal pressure facilitated the migration of the dental implant. The exposure of the implant through the nasal mucosa (14) may cause rhinitis, requiring the resection of the apical part of the implant. However, it has been seen (15) in a 5 to 23 years follow-up study of 4 cases with dental implants that perforated the nasal mucosa, the absence of clinical or radiological complications, even after analysis by nasal endoscopy.

In our case, due to the lack of osseointegration of the implants, the prosthetic rehabilitation was delayed for more than ten months. Wolf *et al*. (10) reported a case of a patient who referred, same our patient, nasal discomfort. The otolaryngologist, suspecting the presence of a dental implant in the nasal fossa, requested a CT scan. Unlike the case published by Meneses *et al*. (11) with the implant placed in the nasal septum or the case presented by Li *et al*. (12) with the migration of the dental implant into the nasal ostium, in our case, as evidenced in the different TC slices, the implant was placed between the nasal mucosa and bone. This situation probably conditioned that in the event of a change in the intranasal pressure, the patient spontaneously evacuated the implant. For patient safety and to avoid further complications, the two remaining implants in the maxilla were surgically removed.

Implant migration into the nasal fossa is a rare complication and it requires extraction by anterior rhinoscopy. To the best of our knowledge, there have been no reports of spontaneously expulsion through the nasal cavity.
